# [Retracted] NG25, an inhibitor of transforming growth factor‑β‑activated kinase 1, ameliorates neuronal apoptosis in neonatal hypoxic‑ischemic rats

**DOI:** 10.3892/mmr.2024.13423

**Published:** 2024-12-23

**Authors:** Hua Wang, Zhong Chen, Yu Li, Qiaoyun Ji

Mol Med Rep 17: 1710–1716, 2018; DOI: 10.3892/mmr.2017.8024

Following the publication of this paper, it was drawn to the Editors’ attention by a concerned reader that certain of the TUNEL assay data shown in Fig. 4B were strikingly similar to data appearing in different form in another article written by different authors at different research institutes that had already been submitted for publication to the journal *Experimental and Therapeutic Medicine* (which has subsequently been retracted). Owing to the fact that these contentious data had already apparently been submitted for publication prior to the receipt of this paper to *Molecular Medicine Reports*, the Editor has decided that this paper should be retracted from the Journal. The authors were asked for an explanation to account for these concerns, but the Editorial Office did not receive a reply. The Editor apologizes to the readership for any inconvenience caused.

